# Inter-Population Movements of Steller Sea Lions in Alaska with Implications for Population Separation

**DOI:** 10.1371/journal.pone.0070167

**Published:** 2013-08-05

**Authors:** Lauri A. Jemison, Grey W. Pendleton, Lowell W. Fritz, Kelly K. Hastings, John M. Maniscalco, Andrew W. Trites, Tom S. Gelatt

**Affiliations:** 1 Division of Wildlife Conservation, Alaska Department of Fish and Game, Douglas, Alaska, United States of America; 2 National Marine Mammal Laboratory, National Marine Fisheries Service, Seattle, Washington, United States of America; 3 Department of Science, Alaska SeaLife Center, Seward, Alaska, United States of America; 4 Marine Mammal Research Unit, Fisheries Centre, University of British Columbia, Vancouver, Canada; Aristotle University of Thessaloniki, Greece

## Abstract

Genetic studies and differing population trends support the separation of Steller sea lions (*Eumetopias jubatus)* into a western distinct population segment (WDPS) and an eastern DPS (EDPS) with the dividing line between populations at 144° W. Despite little exchange for thousands of years, the gap between the breeding ranges narrowed during the past 15–30 years with the formation of new rookeries near the DPS boundary. We analyzed >22,000 sightings of 4,172 sea lions branded as pups in each DPS from 2000–2010 to estimate probabilities of a sea lion born in one DPS being seen within the range of the other DPS (either ‘West’ or ‘East’). Males from both populations regularly traveled across the DPS boundary; probabilities were highest at ages 2–5 and for males born in Prince William Sound and southern Southeast Alaska. The probability of WDPS females being in the East at age 5 was 0.067 but 0 for EDPS females which rarely traveled to the West. Prince William Sound-born females had high probabilities of being in the East during breeding and non-breeding seasons. We present strong evidence that WDPS females have permanently emigrated to the East, reproducing at two ‘mixing zone’ rookeries. We documented breeding bulls that traveled >6,500 km round trip from their natal rookery in southern Alaska to the northern Bering Sea and central Aleutian Islands and back within one year. WDPS animals began moving East in the 1990s, following steep population declines in the central Gulf of Alaska. Results of our study, and others documenting high survival and rapid population growth in northern Southeast Alaska suggest that conditions in this mixing zone region have been optimal for sea lions. It is unclear whether eastward movement across the DPS boundary is due to less-optimal conditions in the West or a reflection of favorable conditions in the East.

## Introduction

Steller sea lions (SSL; *Eumetopias jubatus*) are distributed around the North Pacific rim from central California to northern Japan [1], [2]. In 1997, based on differences in mitochondrial DNA and population trend [3], [4], the National Marine Fisheries Service classified SSLs as two separate distinct population segments (DPS), or stocks, with the dividing line at 144°W longitude. In this paper, we use the terms stock, population, and DPS synonymously. SSLs born at rookeries from central California through southeastern (‘Southeast’) Alaska are considered of eastern DPS (EDPS) origin; those born at rookeries from the central Gulf of Alaska (including Prince William Sound) through Japan are of western DPS (WDPS) origin. These populations have been reproductively isolated for >60,000 years [5] and have been described as separate subspecies, based on both genetic and morphological data [6], [7]. Additional research has described the range west of the Commander Islands, Russia as a potential third stock in Asia [8]. The differentiation between the Asian stock and the WDPS is not as well defined as between the WDPS and the EDPS.

From the 1970s through 2012, the number of SSLs in the WDPS declined dramatically, although the rate and timing of the decline varied spatially [9]–[13]. SSLs in Asian waters have shown similar regional differences in population trend [14]. During this same period, the number of SSLs in the EDPS (Southeast Alaska, British Columbia, Oregon, and Washington) generally increased [15]–[17], except in central California, where the population declined [16]. Prior to the DPS classification and due to the declining total SSL population in Alaska, SSLs were listed as “threatened” under the U.S. Endangered Species Act in 1990 [18]. After the separate DPS had been recognized, the declining WDPS’ status was uplisted to “endangered” under the Endangered Species Act, while the EDPS retained its “threatened” status [19].

There are no physical barriers separating the EDPS and the WDPS that could explain the strong genetic differentiation between the DPS [3], [20], reproductive isolation (possibly for millennia [5]), and the very low rates of exchange of reproductive females across the DPS boundary [3], [21]. SSLs are generally considered to be non-migratory in the sense that not all travel long distances between breeding and non-breeding areas, nor do they move directionally in large groups after the breeding season. But they usually disperse between seasons (*i.e.,* breeding and non-breeding) and might be considered to be short-distance or semi-migrants. On a short time-scale, SSLs forage at sea and return to a central place to breed, provision young, and rest, but they can and do shift their central-place haulout to take advantage of seasonal changes in distribution and abundance of prey resources [22]–[24]. During the breeding season, reproductive adults are strongly associated with rookeries, whereas independent juveniles and non-reproductive adults are not restricted in the same manner. Some animals, in particular males and juveniles, make long-distance movements of >1000 km [21], [25]–[27], however these types of movements are poorly understood and difficult to study, especially over the life span of a SSL.

An early mark-resight study in Alaska found no evidence of adult females giving birth in their non-natal DPS and only one observation of a mature male in its non-natal DPS during the breeding season [21]. At the time of the Raum-Suryan *et al.* [21] study, the closest rookeries within the eastern DPS range (‘East’) and the western DPS range (‘West’) were about 900 km apart (Hazy Islands in the East and Seal Rocks in the West). This began to change in the early 1990s when a new rookery began to form in the northern portion of the East; and another two were initiated a decade later, all three in central and northern Southeast Alaska [16], [17]. These new rookeries are closer to the DPS boundary than any previously established rookery in the East and have reduced the gap between the nearest adjacent rookeries across the DPS boundary to about 640 km.

Recent analyses of mitochondrial DNA samples collected from two of the three newly established rookeries (White Sisters and Graves Rocks) in northern Southeast Alaska found that breeding female founders at these rookeries were derived from both populations, with 30–70% WDPS haplotypes [28], [29]. This suggests that the Graves Rock and White Sisters rookeries have become a reproductive mixing zone for the two DPS and that the reproductive isolation that existed for a long time has diminished.

The goal of our study was to gain further insight into the apparent mixing of the two SSL populations using observations of movements of known individuals that were uniquely marked as pups at rookeries in both the EDPS and WDPS from 2000–2010. Based on resightings of these animals, we determined the annual and seasonal probabilities (by age and sex) of SSLs being in their non-natal DPS. We also explored individual patterns of cross-boundary movements including reproduction in the opposite DPS.

## Methods

### Ethics Statement

Procedures for animal capture, handling, marking, and resighting were approved, and strictly adhered to, under permits issued by the National Marine Fisheries Service to the Alaska Department of Fish and Game (ADF&G, Permit Numbers 358–1564 and 358–1769), the National Marine Mammal Lab (NMML, Permit Numbers 782–1532, 782–1768, 782–1889, and 14326), and the Alaska SeaLife Center (ASLC, Permit Numbers 881-1668-05, 881-1890-02, and 14324), and by ADF&G/Division of Wildlife Conservation’s Animal Care and Use Committee. All branding was performed under isoflurane gas anesthesia, and all efforts were made to minimize suffering. Branding of SSL pups [30], including disturbance to the rookery, has been shown to have little or no effect on their subsequent survival [31], [32].

**Figure 1:**
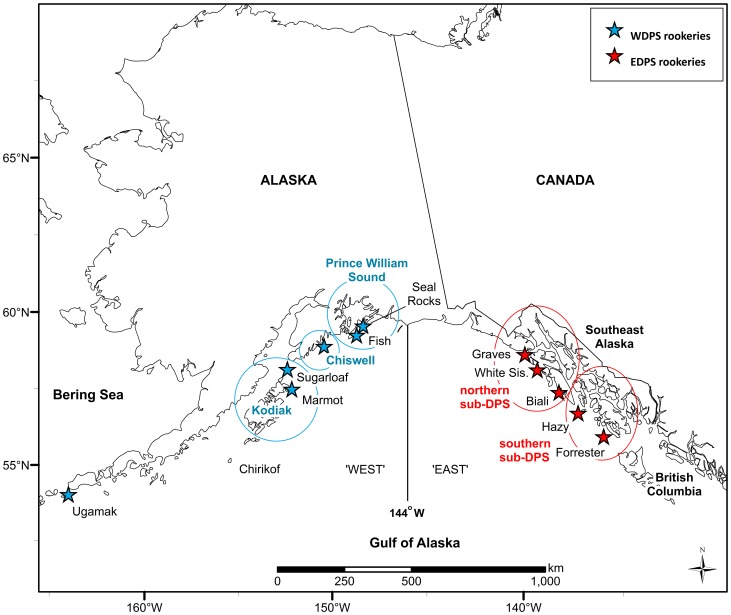
Rookeries where Steller sea lions were branded and region where brand-resight surveys were conducted. Primary study area, from Chirikof Island to Forrester Island, where Steller sea lions in Alaska were branded at natal rookeries in 2000–2010 and resighted from 2000–2012 within the eastern Distinct Population Segment (DPS) and the western DPS. Also shown is the newest rookery (Biali Rocks) that was established in the early 2000s. Sub-regions (circled) within each DPS include: the Kodiak Island, Chiswell Island, and Prince William Sound areas, and northern and southern areas within Southeast Alaska.

ADF&G, the NMML, and the ASLC captured and permanently marked, by hot-branding [30], three to four week old SSL pups on their natal rookeries (n = 10) during 2000–2010 (Figure 1). The primary study area where pups were marked and resight effort was greatest encompassed approximately 850 km southeast of the DPS boundary to Forrester Island and 850 km southwest of the DPS boundary to Chirikof Island. Within this region of the East, pups were branded at four of the five rookeries (no animals were branded at Biali Rocks, the smallest and newest rookery in the EDPS). In the West, sea lions were branded at five of the seven rookeries within the study area (no branding occurred at Chirikof Island or Outer Island). Outside of the core study area (but within Alaska), pups were branded at Ugamak Island in the eastern Aleutian Islands, and were included in our initial analyses. Although marking did not take place at all rookeries across the entire ranges of the two DPS, marking was wide-spread within the primary study area on both sides of the DPS boundary, allowing us to estimate movement probabilities.

In total, 4,187 pups were branded with a unique alpha-numeric mark: 1,995 in Southeast Alaska (EDPS) at four rookeries (Forrester Island, Hazy Islands, White Sisters, Graves Rocks); 1,479 in the central Gulf of Alaska (WDPS) at two rookeries in Prince William Sound (Fish Island and Seal Rocks), two rookeries near Kodiak Island (Marmot Island and Sugarloaf Island), and Chiswell Island; and 713 in the eastern Aleutian Islands (WDPS) at Ugamak Island (Table 1, Figure 1). We analyzed resightings for branded animals except for 15 whose sex was unknown. Although the sex of an animal can be incorrectly identified during handling, our data suggest this was rare in our sample of branded pups. For example, 447 SSLs branded in Southeast Alaska were resighted and photographed at age ≥6 years, when sex is readily evident, at least twice by our most experienced observers. Based on that sample, only 0.029 were determined to have been incorrectly sexed at the time of handling.

**Table 1:** Number of Steller sea lions branded as pups at their natal rookery in the eastern and western DPS within Alaska, 2000–2010.

DPS	Rookery	2000	2001	2002	2003	2004	2005	2007	2008	2009	2010	
East	Forrester Is. (F)^a^		286	141	291	277						995
East	Hazy Is. (H)		213		101		225					539
East	White Sisters (W)			127		94	147					368
East	Graves Rock (V)			50			43					93
	*East total*											1995
West	Seal Rocks (J)		75		100		80					255
West	Fish Is. (E)		32									32
West	Chiswell Is. (E)						26	51	62		60	199
West	Marmot Is. (T)	107		89		75			85		78	434
West	Sugarloaf Is. (X)	151		105		110			93		100	559
West	Ugamak Is. (A)		175		150		200			188		713
	*West total*											2192
	**TOTALS**	**258**	**781**	**512**	**642**	**556**	**721**	**51**	**240**	**188**	**238**	**4187** b

a Letters following the rookery name were included in all brands applied at that location.

b Sex unknown for 15 animals; these animals not included in final analyses.

We conducted annual, dedicated brand-resighting surveys at haulouts and rookeries throughout Alaska and northern British Columbia (Figure 1) during May through August 2000–2012, with greatest survey effort occurring between mid-May and mid-July. Surveys at most sites were conducted from skiffs launched from larger vessels. More intensive land-based surveys were conducted at selected rookeries: Lowrie Island (part of the Forrester Island complex), Marmot Island, Sugarloaf Island, and Ugamak Island, as well as at Round Island (a haulout in the Bering Sea) and through a remote-control video system at Chiswell Island and three neighboring haulouts. Observers in skiffs, on land, and at the ASLC-based remote video receiving station resighted and photographed branded animals. To estimate movement probabilities, we used data collected during years 2000–2010. We only included observations of branded animals with an associated photograph, with the brand identity then confirmed against a master photo library containing all brands. Observations with poor photos and uncertain identification were rejected, even if the field observer felt their visual observation was correct. We were able to include some brands that were difficult to identify in the field but distinctive enough that they could be easily matched from one observation to the next using the photo library. Error associated with misreading brands was eliminated by using only photo-confirmed observations.

We attempted to survey every major haulout and rookery from northern British Columbia through the eastern Aleutian Islands at least once during each breeding season. Extra survey effort was extended to most rookeries and many of the larger haulouts, with 2–3 days dedicated to these larger sites in many years, particularly after 2004. The intensity of survey effort was not uniform across the SSL range because surveys in different parts of Alaska were conducted by personnel from different agencies, and because logistical constraints varied among regions. Weather and logistics also precluded surveys at some sites each year, primarily in the western Gulf of Alaska and the Aleutian Islands. We therefore tried to accommodate the varying effort in our analyses through fitting regional or sub-regional resighting probabilities in models.

In addition to the pups that were branded in our core study area, pups were also branded at natal rookeries in Russia, and in Oregon and California in the U.S., by researchers from Russia and the State of Oregon. These researchers conducted brand-resight surveys at sites in Russian waters and along the U.S. west coast and in southern British Columbia and shared observations and photos. This information, along with results of studies [27], [33] on SSLs marked outside of Alaska and farther from the DPS boundary provides ancillary information to help determine whether the extent of our marking and resighting program was adequate to capture most or all of the inter-DPS movement.

Although effort was much less, we also conducted dedicated brand-resight surveys outside of the breeding season (*i.e.*, August-April). In general, larger scale resight efforts were associated with other SSL projects, including multiple 2–3 week-long capture trips throughout Alaska and observational studies at remote field camps [34], [35]; we conducted skiff surveys throughout Southeast Alaska and received data from quarterly surveys at selected haulouts around Kodiak Island. Remote cameras were used to monitor 1–4 sites in the northern Gulf of Alaska year-round. During both breeding and non-breeding seasons, opportunistic observations with photographs were provided to researchers at ADF&G, NMML, and ASLC by other researchers, tour boat operators, and the general public. Nearly all observations of sea lions used in this study were of animals hauled out on coastal shorelines at known rookeries and haulouts. Although SSLs are, at times, pelagic [36], our study area included only near-shore zones where SSLs spend much of their time, particularly during the breeding season [22], [37], [38], and where they can be observed for marks.

In order to present the most current information on cross-boundary movements, we additionally examined the resight history of only those animals moving from natal to non-natal DPS through August 2012. This required preparing and using a much smaller set of data from 2011 and 2012, and allowed us to present more complete information on reproductive females as well as interesting and unexpected movements of males just reaching maturity during 2011 and 2012.

### Analyses

We tabulated sightings of all branded SSLs into two yearly periods, breeding (May-July) and non-breeding (August-April) seasons. We used these observations to create two resight histories for each SSL, one based only on breeding season observations (hereafter “annual occasions”) and the other with observations from both seasons (hereafter “seasonal occasions”); in the seasonal histories, entries based on observations from breeding and non-breeding seasons alternate.

The long periods we used as resight ‘occasions’, especially in the non-breeding season, could lead to individual heterogeneity in resight probability, which potentially could bias parameter estimates, especially of survival probabilities [39]. However, within these longer periods resight effort generally was clumped in time and space (*i.e.*, effort typically was expended only one or a few times per site), potentially reducing heterogeneity. It is unclear how ‘long occasions’ affect estimates of transition probabilities, but based on our preliminary analyses, transition probability estimates were relatively insensitive to changes in survival estimates.

In a basic resight history, for each occasion a ‘1′ is entered if the animal is observed and a ‘0′ if it is not observed. For our analyses, we were interested in state (*e.g.*, DPS) as well as detection, so 1 s were replaced with a letter indicating which DPS (‘e’ or ‘w’) the SSL was observed in. In addition to estimates of movement between DPSs, we also were interested in knowing whether movement probabilities varied at a finer, sub-DPS (*i.e.*, birth area) scale. We divided the EDPS into two sub-DPS (northern and southern) and the WDPS into three sub-DPS (Prince William Sound, Chiswell, and Kodiak; Figure 1). Because it was computationally prohibitive to include all sub-DPS as geographic states in these models, we constructed additional, modified capture histories to address the fine-scale patterns. These new capture histories, modified from the ones constructed for the full DPS analyses, kept entries for one DPS unchanged, but replaced the entries for the other DPS with 2–3 sub-DPS. In the full DPS resight histories (EW) ‘e’ and ‘w’ (for east and west) are the only geographic states, but in one of the fine-scale histories (snW), for example, ‘e’ is replaced by either an ‘s’ or ‘n’, for the southern or northern parts of the eastern range within Southeast Alaska, respectively; ‘w’ entries are unchanged in these histories. The southern sub-DPS includes animals born at Forrester Island and Hazy Islands whereas the northern sub-DPS includes animals born at White Sisters and Graves Rocks. Similarly, in the other set of sub-DPS resight histories (kcpE), ‘e’ entries are unchanged, but ‘w’ entries are replaced by ‘k’ (Kodiak area, including Marmot and Sugarloaf Islands), ‘c’ (Chiswell Island), or ‘p’ (Prince William Sound, including Seal Rocks and Fish Island) (see Figure 1). This resulted in six sets of capture histories, of which three sets were for annual occasions and three sets were for seasonal occasions; all six sets had the same number of capture histories (*i.e.*, SSLs) differing only in the number of ‘occasions’ (twice as many in the seasonal histories) and how we labeled resight location.

We considered all SSLs used in our analyses to have been ‘observed’ in the breeding season of their birth. Irrespective of how many times a SSL was observed within a single breeding or non-breeding season, a single letter for the area they were observed was entered into the resight history for that season. In a few instances, SSLs were observed in more than one DPS within a single season. For the breeding season, the location of the sighting closest in time to 25 June of that year was used to assign area. For the much longer non-breeding season, the location that maximized the documented movement of that SSL across the DPS boundary was used, based on the logic that the animals had crossed the DPS boundary and we chose the observation that best reflected that movement.

We used multi-state mark-resight models [39] to estimate parameters associated with SSL movements. Multi-state models contain three types of parameters, survival (S_t+1_, the probability that an animal alive at time t, is still alive and in the population subject to observation at time t+1), sighting probability (p_t_, the probability that an animal that is alive and in the population at time t is observed and recorded at that time), and transition probability (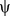
_t_^od^, the probability that an animal at geographic location o (origin DPS or subunit) at time t-1, and that survives to time t, is at location d (destination DPS or subunit) at time t). All of these parameters could be modeled as functions of age, sex, time, natal DPS, and observed region (or sub-region). In this paper we focus on cross-boundary movements of SSLs branded and resighted in Alaska from 2000–2010. Survival has recently been reported for the EDPS-branded SSLs by Hastings *et al.* [40]. Survival of WDPS SSLs has been estimated for those born in Prince William Sound and the Kodiak area (NMML unpublished data) and for juveniles born at Chiswell Island (ASLC unpublished data); manuscripts presenting these results are currently in review.

Because it is recommended that only a small set of biologically plausible models be considered when selecting a model for inference [41], and because it is computationally intensive to fit our models, we considered only nine models of 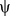
to determine the best age structure (Table 2). We also modeled 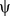 as a function of sex, natal DPS, and season (for the seasonal models). We used only single structures for S and p based on best models from Hastings *et al.* [40]; we modeled S as a function of sex, age (4 classes: 0–1, 1–2, 2–3, 4+), and observation region (*e.g.*, East or West, or sub-region), and modeled p as a function of sex, age (3 classes: 1, 2–4, 4+), observation region, time, and season (seasonal models only) (Table 2). Preliminary analyses indicated strong support for region-specific S. We assumed the S and p model structures were complex enough to account for most of the variation in S and p such that estimates of 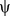 would be unbiased. These sets of 9 models were fit using each of the six sets of capture histories.

**Table 2:** Parameter structures for multistate mark-resight models used to predict the age-specific probabilities of Steller sea lions being present in the opposite DPS with models named after the age structure or 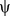, the transition probabilities; the structures of S and p were the same in all models.

	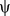 ^od^ structure	age groupings
3a	strat.*to-strat.*sex*b-dps*age3*(season)a, b	1, 2–4,5+
3b	strat.*to-strat.*sex*b-dps*age3*(season)	1–2, 3–4, 5+
4a	strat.*to-strat.*sex*b-dps*age4*(season)	1, 2–4, 5–7, 8+
4b	strat.*to-strat.*sex*b-dps*age4*(season)	1–2, 3–4, 5–7, 8+
4c	strat.*to-strat.*sex*b-dps*age4*(season)	1, 2–4, 5, 6+
4d	strat.*to-strat.*sex*b-dps*age4*(season)	1–2, 3–4, 5, 6+
5a	strat.*to-strat.*sex*b-dps*age5*(season)	1, 2–4, 5, 6–7,8+
5b	strat.*to-strat.*sex*b-dps*age5*(season)	1–2, 3–4, 5, 6–7, 8+
7	strat.*to-strat.*sex*b-dps*age7*(season)	1, 2, 3, 4, 5, 6, 7+
	S structure: sex*age4*strat.	1, 2, 3, 4+
	p structure: strat.*sex*age3*(season)+strat.*time	1, 2–4, 5+

a Stratum (strat.) is the DPS (or sub-DPS) where the sea lion originates, to-stratum (to-strat.) is the DPS where the sea lion moves to in the next interval (year or season), birth-dps (b.-dps) is the natal DPS for a sea lion, and season is an indicator variable for breeding or non-breeding season.

b Season was included only for the seasonal analyses, and not for the breeding-season-only analyses.

We used the programs MARK [42] and RMark [43] to estimate model parameters. After fitting each model series, we selected the model used for inference from among the nine candidate models based on the small-sample corrected Akaike’s Information Criteria (AICc, [41]). We used model averaging [41] to calculate final estimates when the most highly ranked models had AICc values within 2.

For this paper, we were most interested in the transition probabilities, because they could be used to estimate the probability of state occupancy (*i.e.*, the probability of being in a specified state) at a specific age. The letter Ψ also has been used in the mark-resight literature to define state occupancy probability [44]; we thus use a capital Ψ_a_^od^ for occupancy probability and a 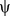
_a_^od^ for transition probabilities, where ‘a’ is age, with o and d as previously defined. Because of our sampling design, our data were not compatible with standard occupancy models [44] and so we could not estimate Ψ_a_^od^ directly. Consequently we calculated Ψ_a_^od^ as derived parameters. Conditional on survival to age ‘a’, Ψ_a_^od^ is calculated as (using an EDPS origin as an example, hence the origin superscript on Ψ_a_^od^ is omitted):
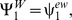
and for age >1



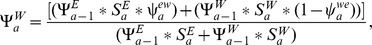
where S_a_^E^ and S_a_^W^ are age-specific survival probabilities for SSLs in the East or West, respectively. We calculated the variances of Ψ_a_^od^ using the delta method [39], converted estimates and their variances to the logit scale, and computed confidence intervals on the transformed quantities, which we then back-transformed to the probability scale to estimate asymmetrical, unbiased confidence intervals [45].

## Results

We observed 61% of the 4172 branded sea lions at least once after the natal period (from birth to 15 August of the year they were born). We recorded 22,059 photo-confirmed sightings of these animals from 2000–2010. Additional photo-confirmed records, obtained from January 2011 through August 2012, of individual animals that crossed to the opposite DPS, were additionally used to describe individual movements.

### Movement Probabilities

The model series for annual occasions (data from the breeding season) estimated age-specific probabilities that a sea lion was in the opposite DPS during the breeding season. Only one Ugamak animal was seen as far east as Kodiak Island (at Latax Rocks north of Kodiak Island, a distance of ∼930 km from Ugamak Island) and none crossed the DPS boundary into the East and therefore, data for Ugamak SSLs were not used to estimate the probabilities we present in this paper. The best models generally had simpler 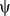-age structure, possibly because of the rapid increase in estimated parameters in the model with more age classes.

During the breeding season, EDPS females were almost never in the West, however males regularly traveled to the West, with the highest probability of occupancy during juvenile years, peaking at age 4 at 0.184, then declining (Table 3). EDPS males from the southern sub-DPS had probabilities of occupancy in the West that were 2–3 times higher after age 1 than those from the northern sub-DPS (Table 3), despite the fact that the northern sub-DPS is closer to the DPS boundary. SSLs from the southern sub-DPS had to either swim through the northern sub-DPS or take a direct pelagic route to the West.

**Table 3:** Estimated annual age- and sex-specific occupancy probabilities for eastern DPS Steller sea lions being in their non-natal DPS during the breeding season; the estimates were derived from the best of 9 models for each set of estimates (see Table 2).

	Ψ^EW^ *		Ψ^nW^		Ψ^sW^	
	(4c [0.47], 3a [0.46])**		(3a [1.00])		(3a [1.00])	
Age	Female	Male	Female	Male	Female	Male
1	0.005	0.082	0.020	0.061	0	0.087
2	0.005	0.128	0.005	0.078	0	0.167
3	0.005	0.150	0.005	0.087	0	0.207
4	0.005	0.184	0.005	0.105	0	0.259
5	0	0.113	0	0.053	0	0.136
6	0	0.059	0	0.032	0	0.087
7	0	0.037	0	0.024	0	0.067
8	0	0.028	0	0.020	0	0.059
9	0	0.024	0	0.019	0	0.056

* Abbreviations for origin and destination areas are: E = EDPS, W = WDPS, n = northern (EDPS), s = southern (EDPS). In the Ψ^od^, the first superscript represents the natal DPS (or sub-DPS) and the second superscript represents the destination DPS.

** Values indicate the best model based on AICc (see Table 2); if more than one model is listed, model averaging was used with models in the order listed, with model weights in brackets.

WDPS males during the breeding season followed the same pattern as EDPS males, with an increasing probability of being in the opposite DPS to age 4 then declining with age. Although WDPS females also regularly traveled to the East, their probability of occupancy was highest at age 1 and declined thereafter (Table 4). WDPS females at age 5 (the major onset of pupping) had a probability of being in the opposite DPS of 0.067, compared to a probability of 0 for same-age EDPS females. In terms of sub-DPS, the probability of being in the East was substantially higher for Prince William Sound SSLs (which is closest to the DPS boundary) compared to low probabilities for Chiswell Island SSLs (which had the smallest sample size of branded animals) but at intermediate levels for more distant Kodiak sub-DPS (Table 4). The probability of Prince William Sound females being in the opposite DPS was particularly high compared to females from all other sub-DPS and was higher than all WDPS males with the exception of those ages 3–7 from Prince William Sound. At age 1, over one-quarter of Prince William Sound females (p = 0.263) moved East, nearly twice the number of males from this sub-DPS (p = 0.138).

**Table 4:** Estimated annual age- and sex-specific occupancy probabilities for western DPS Steller sea lions being in their non-natal DPS during the breeding season; the estimates were derived from the best of 9 models for each set of estimates (see Table 2).

	Ψ^WE^ *		Ψ^pE^		Ψ^cE^		Ψ^kE^	
	(4c [0.47], 3a [0.46])**		(3a [1.00])		(3a [1.00])		(3a [1.00])	
Age	Female	Male	Female	Male	Female	Male	Female	Male
1	0.089	0.082	0.263	0.138	0	0	0.063	0.060
2	0.080	0.103	0.250	0.158	0	0.025	0.045	0.061
3	0.085	0.135	0.179	0.191	0	0.045	0.038	0.072
4	0.079	0.148	0.134	0.218	0	0.062	0.033	0.081
5	0.067	0.090	0.111	0.184	0	0.043	0.028	0.079
6	0.051	0.072	0.096	0.157			0.025	0.077
7	0.042	0.059	0.088	0.105			0.020	0.061
8	0.036	0.050	0.077	0.070			0.016	0.050
9	0.032	0.043	0.072	0.047			0.014	0.042
10	0.029	0.038	0.069	0.031			0.013	0.037

* Abbreviations for origin and destination areas are: E = EDPS, W = WDPS, p = Prince William Sound (WDPS), c = Chiswell (WDPS), k = Kodiak (WDPS). In the Ψ^od^, the first superscript represents the natal DPS (or sub-DPS) and the second superscript represents the destination DPS.

** Values indicate the best model based on AICc (see Table 2); if more than one model is listed, model averaging was used with models in the order listed, with model weights in brackets.

The patterns for the seasonal analyses were similar to the breeding season analyses (Tables 5 & 6), but estimates for breeding season probabilities, which are in both analyses, are not exactly the same between the two sets. This likely is because the additional non-breeding season data affects estimates of 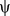. Occupancy probability, Ψ_i_, is calculated using a recursive formula incorporating estimates of 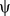
and S for all ages ≤i. In the seasonal models, breeding season occupancy estimates are calculated using 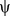 estimates from all preceding seasons, both breeding and non-breeding. Of the two sets of breeding season occupancy estimates, we believe that the estimated probabilities from the breeding-season-only analyses are superior to those from the seasonal analyses that incorporate non-breeding season 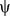 estimates, which were based on less data and are less precise than their counterparts from the breeding season (Appendix S1).

**Table 5:** Estimated seasonal age- and sex-specific occupancy probabilities for eastern DPS Steller sea lions being in their non-natal DPS during breeding (whole number) and non-breeding (+0.5) seasons; the estimates were derived from the best of 9 models for each set of estimates (see Table 2).

	Ψ^EW^ *		Ψ^nW^		Ψ^sW^	
	(4c [0.84])**		(3a [0.99])		(3a [0.99])	
Age	Female	Male	Female	Male	Female	Male
0.5	0.004	0	0.016	0.025	0	0
1.0	0.005	0.098	0.015	0.044	0	0.131
1.5	0.005	0.102	0.014	0.050	0	0.146
2.0	0.005	0.115	0.013	0.050	0	0.182
2.5	0.004	0.117	0.011	0.058	0	0.180
3.0	0.004	0.126	0.010	0.060	0	0.196
3.5	0.004	0.139	0.009	0.073	0	0.212
4.0	0.004	0.155	0.009	0.078	0	0.238
4.5	0	0.153	0.009	0.096	0	0.303
5.0	0	0.118	0	0.055	0	0.115
5.5	0	0.213	0	0.074	0	0.189
6.0	0	0.044	0	0.048	0	0.073
6.5	0	0.148	0	0.067	0	0.151
7.0	0	0.031	0	0.046	0	0.059
7.5	0	0.136	0	0.065	0	0.138
8.0	0	0.028	0	0.045	0	0.054
8.5	0	0.134	0	0.065	0	0.134
9.0	0	0.028	0	0.044	0	0.053
9.5	0	0.134	0	0.064	0	0.132

* Abbreviations for origin and destination areas are: E = EDPS, W = WDPS, n = northern (EDPS), s = southern (EDPS). In the Ψ^od^, the first superscript represents the natal DPS (or sub-DPS) and the second superscript represents the destination DPS.

** Values indicate the best model based on AICc (see Table 2); if more than one model is listed, model averaging was used with models in the order listed, with model weights in brackets.

**Table 6:** Estimated seasonal age- and sex-specific occupancy probabilities for western DPS Steller sea lions being in their non-natal DPS during breeding (whole number) and non-breeding (+0.5) seasons; the estimates were derived from the best of 9 models for each set of estimates (see Table 2).

	Ψ^WE^ *		Ψ^pE^		Ψ^cE^		Ψ^kE^	
	(4c [0.84])**		(3a [1.00])		(3a [1.00])		(3a [1.00])	
Age	Female	Male	Female	Male	Female	Male	Female	Male
0.5	0.019	0.021	0.111	0.068	0	0	0	0.009
1.0	0.069	0.072	0.203	0.139	0	0	0.064	0.069
1.5	0.062	0.081	0.138	0.163	0	0.059	0.062	0.075
2.0	0.074	0.115	0.129	0.176	0	0.070	0.065	0.085
2.5	0.076	0.130	0.114	0.205	0	0.125	0.066	0.103
3.0	0.097	0.163	0.135	0.222	0	0.115	0.071	0.126
3.5	0.088	0.165	0.118	0.231	0	0.163	0.063	0.128
4.0	0.097	0.180	0.137	0.226	0	0.145	0.060	0.131
4.5	0.095	0.182	0.160	0.185	0	0.118	0.065	0.113
5.0	0.076	0.092	0.093	0.156	0	0.094	0.043	0.109
5.5	0.104	0.093	0.121	0.129	0	0.082	0.050	0.096
6.0	0.049	0.083	0.073	0.099			0.036	0.086
6.5	0.077	0.084	0.105	0.082			0.044	0.076
7.0	0.037	0.075	0.064	0.063			0.032	0.070
7.5	0.065	0.076	0.096	0.052			0.040	0.062
8.0	0.031	0.069	0.058	0.040			0.030	0.058
8.5	0.060	0.070	0.092	0.033			0.038	0.051
9.0	0.028	0.064	0.056	0.025			0.028	0.050
9.5	0.057	0.064	0.089	0.021			0.037	0.044
10.0	0.027	0.059	0.054	0.016			0.028	0.045
10.5	0.056	0.060	0.088	0.013			0.036	0.039

* Abbreviations for origin and destination areas are: E = EDPS, W = WDPS, p = Prince William Sound (WDPS), c = Chiswell (WDPS), k = Kodiak (WDPS). In the Ψ^od^, the first superscript represents the natal DPS (or sub-DPS) and the second superscript represents the destination DPS.

** Values indicate the best model based on AICc (see Table 2); if more than one model is listed, model averaging was used with models in the order listed, with model weights in brackets.

**Figure 2:**
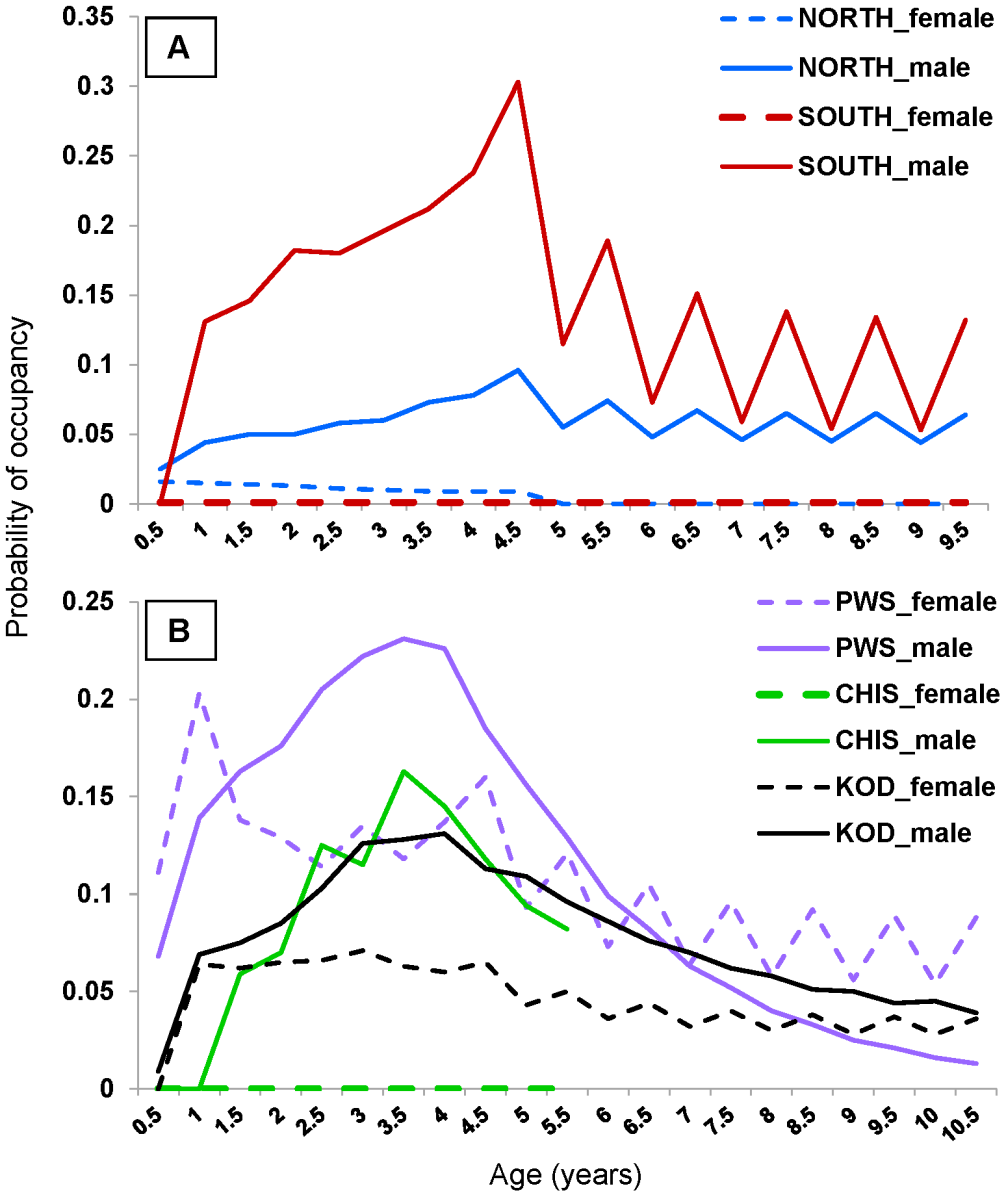
Occupancy probabilities of Steller sea lions being in non-natal DPS during breeding and non-breeding seasons. Estimated seasonal age- and sex-specific occupancy probabilities for sea lions being in their non-natal Distinct Population Segment (DPS) during breeding (whole number) and non-breeding (+0.5) seasons. Dashed lines represent females and solid lines represent males. Graph A shows estimated probabilities of occupancy in the opposite DPS for EDPS sea lions from the northern (NORTH) and southern (SOUTH) sub-DPS within Southeast Alaska. Graph B shows estimated probabilities of occupancy in the opposite DPS for WDPS sea lions from sub-regions Kodiak Island (KOD), Chiswell Island (CHIS), and Prince William Sound (PWS) within the central Gulf of Alaska; see Figure 1.

Of particular note in the seasonal analyses is that the probabilities of EDPS males being in the opposite DPS were nearly always higher in the non-breeding season―a pattern much more pronounced among southern sub-DPS males >4 years (Table 5, Figure 2). Prince William Sound females at ages 4+ showed a similar pattern as southern sub-DPS males (though not quite as exaggerated; Table 6, Figure 2), suggesting that these SSLs in particular disperse seasonally with a substantial proportion crossing the DPS boundary, and that many of them return to or visit their natal DPS during the breeding season. Older Prince William Sound and Kodiak males did not follow this seasonal pattern of higher occupancy during the non-breeding season, but instead, occupancy declined steadily after peaking at age 4 (Table 6, Figure 2).

### Individuals Crossing the DPS Boundary

We observed 118 EDPS SSLs (6% of those branded in Southeast Alaska) in the West; only two of these were females. One female returned to the East and eventually pupped at her natal rookery, then revisited the West with her dependent (*i.e.*, still suckling) juvenile one year later. The second female was seen just once when 11 months old.

In contrast to the EDPS sea lions, 89 WDPS animals (6% of those branded in the central Gulf of Alaska) were observed in the East, of which 35 (39%) were females. Nine WDPS females gave birth at rookeries in the East, at either Graves Rock or White Sisters, in the northern sub-DPS (Tables 7 & 8). Eight of these nine females have never been seen in the West (or in one case, seen at the DPS boundary – Cape St. Elias) after they were first seen in the East. The remaining female, T23 born at Marmot Island, pupped in the East (Graves Rock) at age 5 then subsequently pupped in the West (Sugarloaf Island) at age 8; she has not been seen since. To date, we have strong evidence that at least five of these nine WDPS females have permanently emigrated. Each has been resighted multiple times each year during the breeding season since first arriving in the East (up to ages 7–9), and three have pupped at least twice in the East. In contrast, despite greater numbers of EDPS females branded at Forrester Island and Hazy Islands (in the southern sub-DPS), and the closer proximity of these rookeries to Graves Rock and White Sisters (in the northern sub-DPS), only seven EDPS females from these two southern-most rookeries in Alaska have pupped at Graves Rocks or White Sisters (Table 8).

**Table 7:** Female Steller sea lions born in the western DPS that gave birth in the eastern DPS within Alaska, 2000–2012.

Natal rookery (sub-DPS)	Brand	Birth Year	Pupping location and yearin eastern DPS	Comment
Seal Rocks (WDPS-p) a	J144	2003	Graves: 2008, 2010	Never seen WDPS
Seal Rocks	J159	2003	Graves: 2008	Never seen WDPS
Seal Rocks	J233	2005	Graves: 2010, 2011	1^st^ sighting at C. St. Elias (DPS boundary) age 4;seen only EDPS since then
Seal Rocks	J252	2005	Graves: 2012	Not seen WDPS since 2 mos. of age
Marmot (WDPS-k) b	T23	2000	Graves: 2005, Sugarloaf c: 2008	Age 1–6 EDPS, Age 7–8 WDPS; last seen 2008
Marmot	T202	2004	Graves: 2009–2011	Not seen WDPS since 2 mos. Age
Marmot	T246	2004	Graves: 2009	Not seen WDPS since 2 mos. Age
Sugarloaf (WDPS-k)	X144	2000	White Sisters: 2008	Never seen WDPS; gaps in resight history
Sugarloaf	X321	2004	White Sisters: 2009, 2012	Never seen WDPS

a WDPS-p = Prince William Sound sub-region within WDPS.

b WDPS-k = Kodiak sub-region within WDPS.

c Sugarloaf Island is located in the western DPS.

**Table 8:** Number of female Steller sea lions branded at each rookery in Alaska, number seen in the non-natal DPS, number subsequently giving birth in the northern sub-DPS (Graves Rock and White Sisters), and distance from natal rookery to pupping rookery.

Natal DPS (sub-DPS)	Natal Rookery	# females branded^a^	# females in non-natal DPS	# pupped atGraves	Distance to Graves (km)^b^	# pupped at White Sisters	Distance to White Sisters (km)^b^
EDPS (s)^c^	Forrester	449	0	1	435	2	360
EDPS (s)	Hazy Is	251	0	1	300	3	225
WDPS (p)^d^	Seal/Fish	112	17	4	640	0	715
WDPS (k)^e^	Marmot Is	180	11	3	980	0	1060
WDPS (k)	Sugarloaf Is	212	7	0	955	2	1030

a Number of females branded through 2008, therefore of reproductive age (≥4 years) in 2012.

b Distance measured by following coast, not a straight-line distance; rounded to nearest 5 km.

c E = EDPS (s) = southern sub-DPS.

d WDPS (p) = Prince William Sound sub-DPS.

e WDPS (k) = Kodiak sub-DPS.

Eighty-six percent of WDPS females observed in the East were seen in multiple years. Of these 30 animals, 19 returned to their natal DPS where at least 14 eventually had a pup (including T23, who gave birth in both DPS). It was common (>60%) for the females that returned to their natal DPS to later revisit the East, and in some cases were observed nursing their juveniles in northern Southeast Alaska. At least one WDPS female made multiple trips between the East and her natal rookery when she reached maturity. At ages 5, 8, and 9, J141 hauled out at Gran Point (northern Southeast Alaska) in April-May, and was later seen in July of those years at Seal Rocks (Prince William Sound).

Overall, 116 EDPS males and 54 WDPS males were observed across the DPS boundary; some of these SSLs were resighted just once in their lives. Of the males that were resighted in >1 year, 43% of EDPS males and 68% of WDPS males were seen multiple years in their non-natal DPS. The youngest male we have seen at a rookery holding a territory containing adult females for >5 days was 8 years old (ADF&G unpublished data). By including resightings from 2011 and 2012, we increased the numbers of males resighted at ≥8 years of age from 33 to 54 animals.

Thirty-six EDPS males that traveled to the opposite DPS were resighted to ≥8 years old, only one of which was seen exclusively in the West since first arriving at age 4. This male was photographed at haulouts in the Bering Sea four times and in the Prince William Sound and Kenai Fjords region three times. The remaining bulls returned to their natal DPS where 15 held territories at their natal rookery and one held a territory at an adjacent rookery.

For WDPS males that crossed the DPS boundary, 18 were resighted to ≥8 years old. Of those males, two may have emigrated permanently to the East. T22 was seen exclusively in the East over multiple years―hauled out at Graves Rock during the breeding season from ages 7–10 where he held a territory for at least one day. A second male, X207, was documented in the East multiple times from ages 1–9.8. Fifteen mature WDPS males seen in the East returned to their natal DPS where 10 eventually defended a territory (although in some cases this was late in the breeding season). Of these, 60% held a territory at their natal rookery. The remaining male, T25 born in 2000 at Marmot Island, defies the pattern, as he was resighted at Graves Rock at 3 years old―returned to the West and was seen at four haulouts in the northern Gulf of Alaska―before arriving at his natal rookery at age 7. At age 8, he held a territory at Marmot Island for at least one day in late July. The following year T25 was at Graves Rocks in early July, then two weeks later moved to Sugarloaf Island where he hauled out with a small group of adult females and pups. At ages 10–12, he was at Graves Rock during the peak of the breeding season where he has held a territory for the last two years (2011 and 2012).

### Long Distance Movements

We present the history of two males (H183 and F2102) that crossed the DPS boundary as examples of long distance movements between breeding and non-breeding seasons. Surveys during the non-breeding season in the West were rare outside of the Chiswell and Cape Resurrection area, especially so in the Aleutian Islands and the Bering Sea. It was therefore fortuitous that we documented these movements.

H183, a male born in 2001 at Hazy Islands in the East, was present multiple times at haulouts and rookeries in northern Southeast Alaska from ages 0.8–4.7. At ages 6 and 6.2, he was observed at Marmot Island and Rootok Island (eastern Aleutian Islands) in the West. In July 2010, at age 9, H183 was a territorial bull at his natal rookery. In December of that year he returned to the West and was seen at St. Lawrence Island in the northern Bering Strait, a one-way distance (assuming near-shore travel) of ∼3,500 km. The following July (2011), H183 was in Southeast Alaska (115 km north of Hazy Islands) and in 2012 he again held a territory at Hazy Islands. To our knowledge, this is the longest documented movement of a Steller sea lion.

**Figure 3:**
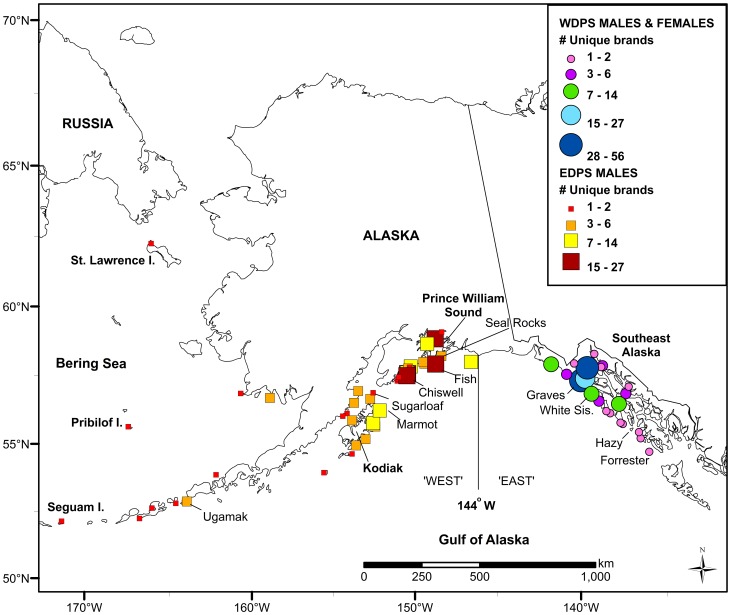
Location and number of individual Steller sea lions resighted in their non-natal DPS. Squares show location of eastern Distinct Population Segment (DPS) males in the West and circles represent western DPS male and female sea lion locations in the East. Not included on map are observations of a male WDPS SSL that was resighted in northern Washington.

F2102, a male born at Forrester Island in 2002, was documented at two sites in northern Southeast Alaska at ages 1 and 4. At age 6 he visited Forrester Island (20 June), then moved to Marmot Island (23 July). At ages 7 and 8 he visited rookeries in the East (two each year) during the breeding season. In July 2011 at age 9, F2102 was again at Forrester Island, and later at Seguam Island (central Aleutian Islands - WDPS) in March 2012. He returned to Forrester Island as a territorial bull in June 2012, a round-trip distance of ∼6,000 km. Our data suggest that these very long distance movements are more common among EDPS males than WDPS males (Figure 3). The greatest known distance a WDPS SSL traveled across the DPS boundary was ∼2,000 km. The majority (>85%) of all WDPS animals observed in the East were at locations in the northern region of Southeast Alaska, whereas EDPS males moved more broadly throughout the West (Figure 3).

## Discussion

We documented the regular movement of Steller sea lions from both the EDPS and WDPS across the defined DPS boundary. Overall, the probability of occupancy in the opposite DPS was highest for sea lions born in Prince William Sound and EDPS males from the southern sub-DPS (born at Forrester and Hazy Islands). Movement of EDPS females to the West was very rare, yet the probability of WDPS females being in the East was 0.067–0.089 at ages 1–5, and remained moderately high (0.069) for Prince William Sound-born females even to age 10. Movements of EDPS and WDPS males followed a similar pattern during the breeding and non-breeding season, with the probability of being in the opposite DPS increasing as they aged to 4–5, then declining afterwards. However, EDPS males aged 5+, were ∼2–4 times more likely to be in the West during the non-breeding season compared to being there during the breeding season―whereas WDPS males had similar probabilities for both seasons. Seasonal movement to the East during the non-breeding season was also evident at older ages for females born in Prince William Sound, which was much higher than for the males born in Prince William Sound.

We have strong evidence that some females from three western rookeries have permanently emigrated to the East, and are reproducing at White Sisters and Graves Rocks, the two rookeries in the mixing zone region of northern Southeast Alaska. It also is notable that more marked WDPS females gave birth at mixing zone rookeries than have females born at Hazy Island and Forrester Island, rookeries that are closer to the mixing zone and in the same DPS. This highlights the skewed movement pattern and important contribution of WDPS females to the formation and growth of the mixing zone rookeries, particularly by females born in Prince William Sound.

Some inter-DPS movements were of short duration, with individuals returning to their natal DPS the following season or year. Others fit a pattern of longer-term temporary residency where individuals were seen multiple years in the range of the opposite DPS then subsequently returned to their natal DPS prior to becoming reproductive. Fifty-four breeding-age males were seen in the opposite DPS during this study; most (94%) eventually returned to their natal DPS where half have defended territories on rookeries, although in some cases this was late in the breeding season when rookery structure begins to break down. Our data suggest that two WDPS males may have permanently moved to the opposite DPS, but we have not observed either male successfully defending a territory for more than one day. A third WDPS male traveled repeatedly across the DPS boundary, at least temporarily holding a territory at two rookeries in the West in consecutive years (ages 8–9) before holding a territory at a rookery in the East for multiple days at ages 11–12 and likely breeding there.

Males of other Otariid species are known to make long-distance movements outside the breeding season (*e.g.*, Antarctic fur seals, *Arctocephalus gazella*, [46]; Australian fur seals, *Arctocephalus pusillus doriferus*, [47]; northern fur seals, *Callorhinus ursinus,* [48]; and New Zealand sea lions, *Phocarctos hookeri,* [49]). Long-distance movements by juvenile and male SSLs have also been documented [21], [25], [27]. Along the U.S. West Coast, adult male SSLs are most abundant in Oregon and northern California during summer but few are present at haulouts during winter months. Males disperse north in late summer and fall and have been observed moving back into Oregon and California in mid-April [27], a pattern that is also seen in California sea lions (*Zalophus californianus*) along this coast [50], [51].

Our study supports and expands on the findings of other pinniped movement studies. We document long-distance, round trip movements of reproductive SSL males between their natal rookery during the breeding season and distant haulouts in their non-natal DPS during the non-breeding season. Some of these are 6,000–7,000 kms round-trip, assuming SSL paths of travel were near-shore. Exactly what drives such long distance movements is unknown. During the breeding season, reproductive adults are strongly associated with rookeries, however once males leave the rookery they search for prey to replenish depleted reserves. During winter surveys, we have noted that adult male sea lions are noticeably absent at most haulouts we visit. SSLs are known to seek out seasonally abundant prey [23], [24], suggesting that males likely make short and long-distance movements during the non-breeding season to take advantage of abundant prey resources.

Harlin-Cognato *et al.* [5] estimated that the EDPS and WDPS had been reproductively isolated for >60,000 years, a sufficient time even for morphological differences to develop [6]. Pitcher *et al.* [16] indicated that there was a northward shift in both the distribution of rookeries and the number of animals in the EDPS throughout the 1900s, reducing the gap between the DPS. With the establishment of the White Sisters rookery in the early 1990s and the Graves Rock rookery in 1999 [15], [17] the gap between DPS rookeries narrowed and a mixing zone formed. Several genetic analyses [3], [8], [52] have documented significant differences between EDPS and WDPS sea lions. However, these studies did not have samples from Graves Rock, the mixing zone rookery with the greatest proportion of WDPS haplotypes [28], [29], and the results from the White Sisters rookery, which also had a substantial proportion of WDPS haplotypes, had the most anomalous results for any of the EDPS rookeries (Personal communication from J. W. Bickham, Battelle Memorial Institute, Huston, Texas, USA, March 2011). It is possible that the reproductive mixing zone likely formed after 1990, and its presence was not evident in the genetics studies that had few or no samples from this area.

Earlier mark-resight studies found no evidence of adult females giving birth in their non-natal DPS and generally little interchange across the DPS boundary [21]. Overall, 1.4% of males and 0.3% of females branded at Marmot Island in 1986–1987 were seen in the East; higher percentages of Forrester Island animals (4.6% males, 1.4% females) branded in 1994–1995 were seen in the West. At the time of branding during the Raum-Suryan *et al.* [21] study, there were two established rookeries in the southern part of Alaska and the newly formed rookery at White Sisters; Graves Rock had not yet emerged as a rookery [17]. Thus, the rookeries where we documented the greatest cross-boundary movement, from Prince William Sound in the West to the mixing zone rookeries in the East, were either not represented in the study (Prince William Sound rookeries) or were still emerging as rookeries (Grave Rock and White Sisters). Our results indicate permanent WDPS emigration to the East by females that subsequently reproduce at mixing zone rookeries, which closely matches recent mtDNA results [28], [29]. We only observed two temporary movements of EDPS females to the West, also consistent with results of the genetic analyses.

Although the overall population dynamics of the EDPS and WDPS have differed over the last 30 years, not all areas within each DPS have shared the same population dynamics [2], [12], [14], [40], [53]–[55]. In the West, SSL counts declined throughout the range, but the rate and timing of the decline differed spatially. Three new rookeries formed in the central and western Gulf of Alaska and one in the central Aleutians, but four sites in the Aleutians and one site in the Bering Sea ceased functioning as rookeries; reproduction declined severely at other western rookeries [13]. In the East, SSL counts increased at virtually all sites and new rookeries were formed, except at the southern-most portion of the sea lion range in central California [16]. Population growth at Forrester Island, the largest rookery in the EDPS, slowed and eventually stabilized during this period; Calkins *et al.* [15] suggested this could be due to space or food limitations, perhaps resulting in animals from this rookery moving north to colonize the Hazy Islands and White Sisters rookeries. The northern-most area within the defined range of the EDPS has experienced the most rapid population growth and highest survival yet estimated for this species [17], [40].

The new rookeries established in the mixing zone formed in the 1990s following a period when conditions in the West were poor, as evidenced by lower survival and reproduction [54]–[56] and conditions in the mixing zone were presumably optimal for survival and potentially reproduction. A climate shift in 1976–1977, causing a reduction in important SSL prey species, may have been the driving force behind lower survival and reproduction in SSLs in the Gulf of Alaska [57]. Hazardous conditions such as mortality incidental to commercial fisheries, commercial hunting, legal and illegal shooting [58] and/or reduced prey availability in the Gulf of Alaska in the past may have favored female dispersal, particularly to the east/south. As the genetic structure of the population changed over time, with dispersing females having greater survival and reproductive success than those in their natal DPS, it appears that a pattern of female movement from West to East was established. Several lines of evidence support the idea that favorable environmental conditions have developed for SSLs within the mixing zone region of northern Southeast Alaska. These include emigration of WDPS females to the East and their reproduction at mixing zone rookeries, while EDPS females seldom travel to the West, the more modest movements of northern sub-DPS males to the West compared to their southern sub-DPS counterparts, despite the northern sub-DPS’ closer proximity to the West, recent studies documenting high survival rates of sea lions that are born in or move to northern Southeast Alaska [based on the same cohorts of branded SSLs from Southeast Alaska whose movements we’ve described in this paper; 40], and a rapidly increasing population [17].

During the 1970s–1980s, a new rookery emerged (or was re-established – pupping may have occurred more than a century earlier) on Medny Island, one of the Commander Islands in Russia [14]. Genetic results indicate that founders of this rookery were from the WDPS [8]. Thus, the establishment of the Medny rookery was the result of a westward movement by WDPS SSLs that narrowed the gap between breeding sites in the WDPS and the proposed Asian stock, much as the formation of the mixing zone rookeries within the EDPS reduced the distance between the nearest rookeries in the EDPS and WDPS. Although there is seasonal westward movement of Medny Island animals to rookeries on the Kamchatka Peninsula (within the proposed Asian stock), there is apparently little or no eastward movement by SSLs born at Asian stock rookeries to Medny Island (Personal communication from V. N. Burkanov, North Pacific Wildlife Consulting, Seattle, Washington, USA, April 2013). Within the proposed Asian stock, a new and rapidly growing rookery was established during the late 1980s–1990s at Tuleny Island near Sakhalin Island, largely the result of emigration of females from Iony Island, ∼900 km to the north (Personal communication from V. N. Burkanov, North Pacific Wildlife Consulting, Seattle, Washington, USA, April 2013). These movements at the western-most edge of the WDPS and within the Asian stock suggest that asymmetric SSL cross-boundary movements and the eventual establishment of new breeding sites is not unique to our region of study and may be an adaptation of this species to changes in their environment.

For much of the past 15 years, SSL management in the U.S. has been structured by the division of the population into two DPS with the boundary at 144°W longitude [4]. This division was based on the phylogenetic method and focused on differing genetics and population dynamics data [3], [4]. However, with the establishment of new rookeries in northern Southeast Alaska, additional genetic data and analyses (*e.g.*, [28], [29]), and the movement data we present, this boundary is no longer as clearly demarcated as it was in the past. These findings highlight the evolving dynamics of the growing SSL populations in both DPSs in the northern Gulf of Alaska.

For our study, we did not have branded SSLs from every rookery throughout each DPS, which could weaken our results if animals born at rookeries far from the DPS boundary regularly moved to the opposite DPS. However we do not think that this is the case. SSLs branded at Ugamak Island, the rookery with marked SSLs farthest west from the DPS boundary, rarely traveled east; the greatest eastward movement by a Ugamak animal was 930 km, still 500 km west of the DPS boundary. Nine male SSLs branded in the southern part of the East in California or Oregon were seen in the West (Personal communication from B. E. Wright, Oregon Department of Fish and Wildlife, Corvallis, Oregon, USA, January 2013), the farthest traveling nearly 3,000 km from its natal rookery, similar to long distance movements of southern sub-DPS males. By comparison, most WDPS SSLs that moved to the East remained in northern Southeast Alaska; only one WDPS animal has been recorded south of Alaska, a 1 year old male from Seal Rocks that traveled ∼2,000 km to northern Washington. No SSLs were branded in British Columbia, yet there are several large rookeries there. Given the long distance movements demonstrated by southern sub-DPS males and males from California/Oregon, we could be underestimating overall East to West movement by EDPS animals, since it is reasonable to assume that some males from British Columbia travel north and occasionally cross the DPS boundary.

Our results support recent genetic analyses that have shown the establishment of SSL rookeries in the mixing zone region of northern Southeast Alaska was due in part to emigration of WDPS SSLs. Although the majority of the cross-boundary movement appears temporary with individual sea lions returning to their natal DPS when they reach breeding age, the collective evidence clearly documents permanent emigration that is biased to WDPS females moving to the East.

WDPS animals began moving to the East following a steep population decline, leaving a region of relatively low density for a region where abundance was at an all-time high [16]. This eastward movement suggests that for whatever reasons (*e.g.*, increased predation, reductions in quality or quantity of available prey), the ecosystem in the central Gulf of Alaska had changed substantially. It also suggests that conditions were far more favorable for SSLs in northern Southeast Alaska, although there is also evidence that conditions have improved somewhat in the Gulf of Alaska based on higher natality and survival during the 2000s ([59], ASLC unpublished data, NMML unpublished data) and short-term population trends in the region have been positive [13]. Whether the continued eastward movement we documented is due to poorer conditions in the central Gulf of Alaska compared to northern Southeast Alaska, or a new behavioral pattern that has been established, especially by WDPS females, is unknown.

## Supporting Information

Appendix S1 Standard Errors and approximate 95% Confidence Intervals for occupancy estimates from Tables 3–6; all abbreviations and formatting are the same as for Tables 3–6.
(DOCX)
