# Supplementary material for: Inter-Population Movements of Steller Sea Lions in Alaska with Implications for Population Separation
Source: PLoS One. 2013 Aug 5;8(8):e70167. doi: 10.1371/journal.pone.0070167 (PMC3734025; doi:10.1371/journal.pone.0070167)
Supplement: Appendix S1 — Standard Errors and approximate 95% Confidence Intervals for occupancy estimates from Tables 3–6; all abbreviations and formatting are the same as for Tables 3–6. (DOCX) [file pone.0070167.s001.docx]

Appendix S1. Standard Errors and approximate 95% Confidence Intervals for occupancy estimates from Tables 3-6; all abbreviations and formatting are the same as for Tables 3-6.

Table S1 (refer to Table 3).

|  | Ψ^EW*^ | |  | Ψ^nW^ | | Ψ^sW^ | |  |  |
| --- | --- | --- | --- | --- | --- | --- | --- | --- | --- |
|  | **(4c, 3a)**** | |  | **(3a)** | | **(3a)** | |  |  |
| **Age** | **Female** | **Male** |  | **Female** | **Male** | **Female** | **Male** |  |  |
| 1 | 0.004 0.001-0.020 | 0.016 0.055-0.120 |  | 0.020 0.003-0.131 | 0.024 0.028-0.129 | -- | 0.019 0.056-0.133 |  |  |
| 2 | 0.004 0.001-0.021 | 0.015 0.100-0.161 |  | 0.005 0.001-0.034 | 0.020 0.046-0.128 | -- | 0.022 0.127-0.215 |  |  |
| 3 | 0.003 0.001-0.018 | 0.019 0.118-0.191 |  | 0.005 0.001-0.034 | 0.022 0.052-0141 | -- | 0.030 0.154-0.272 |  |  |
| 4 | 0.003 0.001-0.018 | 0.024 0.141-0.237 |  | 0.005 0.001-0.034 | 0.028 0.062-0.173 | -- | 0.039 0.189-0.343 |  |  |
| 5 | -- | 0.028 0.069-0.180 |  | -- | 0.015 0.032-0.090 | -- | 0.025 0.094-0.192 |  |  |
| 6 | -- | 0.015 0.036-0.096 |  | -- | 0012 0.015-0.066 | -- | 0.022 0.052-0.140 |  |  |
| 7 | -- | 0.015 0.017-0.079 |  | -- | 0.012 0.009-0.061 | -- | 0.020 0.037-0.119 |  |  |
| 8 | -- | 0.016 0.009-0.082 |  | -- | 0.012 0.006-0.062 | -- | 0.020 0.031-0.111 |  |  |
| 9 | -- | 0.016 0.006-0.089 |  | -- | 0.012 0.005-0.063 | -- | 0.019 0.028-0.109 |  |  |

Table S2 (refer to Table 4).

|  | Ψ^WE*^ | |  | Ψ^pE^ | | Ψ^cE^ | | Ψ^kE^ | |
| --- | --- | --- | --- | --- | --- | --- | --- | --- | --- |
|  | **(4c, 3a) **** | |  | **(3a)** | | **(3a)** | | **(3a)** | |
| **Age** | **Female** | **Male** |  | **Female** | **Male** | **Female** | **Male** | **Female** | **Male** |
| 1 | 0.018 0.059-0.132 | 0.018 0.053-0.123 |  | 0.065 0.157-0.407 | 0.045 0.071-0.252 | -- | -- | 0.018 0.036-0.108 | 0.017 0.033-0.104 |
| 2 | 0.014 0.056-0.112 | 0.017 0.075-0.140 |  | 0.056 0.156-0.374 | 0.039 0.095-0.249 | -- | 0.030 0.002-0.233 | 0.014 0.025-0.081 | 0.017 0.035-0.104 |
| 3 | 0.016 0.058-0.123 | 0.023 0.096-0.187 |  | 0.041 0.111-0.275 | 0.045 0.118-0.294 | -- | 0.055 0.004-0.368 | 0.012 0.021-0.071 | 0.025 0.036-0.138 |
| 4 | 0.018 0.050-0.122 | 0.029 0.100-0.213 |  | 0.035 0.079-0.217 | 0.061 0.123-0.361 | -- | 0.076 0.005-0.455 | 0.012 0.016-0.066 | 0.034 0.034-0.179 |
| 5 | 0.019 0.038-0.116 | 0.033 0.043-0.181 |  | 0.027 0.068-0.175 | 0.051 0.104-0.306 | -- | 0.053 0.004-0.354 | 0.010 0.014-0.057 | 0.031 0.036-0.165 |
| 6 | 0.018 0.025-0.102 | 0.032 0.030-0.164 |  | 0.028 0.053-0.168 | 0.056 0.076-0.297 |  |  | 0.011 0.011-0.057 | 0.033 0.033-0.172 |
| 7 | 0.019 0.017-0.099 | 0.032 0.020-0.163 |  | 0.030 0.041-0.163 | 0.046 0.043-0.233 |  |  | 0.010 0.007-0.054 | 0.029 0.024-0.149 |
| 8 | 0.019 0.012-0.101 | 0.033 0.013-0.171 |  | 0.031 0.034-0.163 | 0.037 0.024-0.187 |  |  | 0.010 0.005-0.054 | 0.027 0.017-0.138 |
| 9 | 0.020 0.009-0.106 | 0.034 0.009-0.185 |  | 0.031 0.030-0.163 | 0.030 0.013-0.154 |  |  | 0.010 0.003-0.056 | 0.026 0.013-0.133 |
| 10 | 0.021 0.007-0.112 | 0.035 0.006-0.202 |  | 0.032 0.027-0.163 | 0.023 0.007-0.127 |  |  | 0.010 0.003-0.059 | 0.025 0.009-0.132 |

Table S3 (refer to Table 5).

|  | Ψ^EW*^ | |  | Ψ^nW^ | | Ψ^sW^ | |  |  |
| --- | --- | --- | --- | --- | --- | --- | --- | --- | --- |
|  | **(4c) **** | |  | **(3a)** | | **(3a)** | |  |  |
| **Age** | **Female** | **Male** |  | **Female** | **Male** | **Female** | **Male** |  |  |
| 0.5 | 0.003 0.001-0.017 | -- |  | 0.011 0.004-0.061 | 0.023 0.004-0.135 | -- | -- |  |  |
| 1.0 | 0.004 0.001-0.020 | 0.016 0.070-0.134 |  | 0.011 0.004-0.060 | 0.019 0.018-0.100 | -- | 0.023 0.092-0.181 |  |  |
| 1.5 | 0.004 0.001-0.020 | 0.015 0.076-0.136 |  | 0.010 0.003-0.054 | 0.017 0.025-0.096 | -- | 0.024 0.106-0.199 |  |  |
| 2.0 | 0.004 0.001-0.020 | 0.015 0.089-0.147 |  | 0.009 0.003-0.050 | 0.014 0.029-0.085 | -- | 0.026 0.136-0.238 |  |  |
| 2.5 | 0.003 0.001-0.017 | 0.015 0.091-0.150 |  | 0.008 0.003-0.044 | 0.016 0.033-0.100 | -- | 0.026 0.133-0.238 |  |  |
| 3.0 | 0.003 0.001-0.015 | 0.017 0.097-0.162 |  | 0.007 0.002-0.039 | 0.017 0.034-0.104 | -- | 0.030 0.143-0.261 |  |  |
| 3.5 | 0.003 0.001-0.015 | 0.018 0.107-0.179 |  | 0.007 0.002-0.038 | 0.021 0.041-0.127 | -- | 0.032 0.156-0.282 |  |  |
| 4.0 | 0.003 0.001-0.015 | 0.021 0.119-0.200 |  | 0.007 0.002-0.037 | 0.023 0.043-0.136 | -- | 0.036 0.174-0.316 |  |  |
| 4.5 | -- | 0.021 0.116-0.200 |  | 0.007 0.002-0.037 | 0.026 0.034-0.089 | -- | 0.039 0.236-0.384 |  |  |
| 5.0 | -- | 0.023 0.080-0.171 |  | -- | 0.014 0.034-0.089 | -- | 0.022 0.078-0.166 |  |  |
| 5.5 | -- | 0.033 0.155-0.286 |  | -- | 0.020 0.043-0.125 | -- | 0.030 0.137-0.256 |  |  |
| 6.0 | -- | 0.013 0.025-0.077 |  | -- | 0.015 0.026-0.089 | -- | 0.019 0.043-0.122 |  |  |
| 6.5 | -- | 0.030 0.098-0.218 |  | -- | 0.021 0.036-0.124 | -- | 0.031 0.100-0.222 |  |  |
| 7.0 | -- | 0.010 0.157-0.060 |  | -- | 0.015 0.023-0.090 | -- | 0.018 0.032-0.106 |  |  |
| 7.5 | -- | 0.032 0.084-0.212 |  | -- | 0.022 0.033-0.125 | -- | 0.032 0.087-0.212 |  |  |
| 8.0 | -- | 0.010 0.014-0.055 |  | -- | 0.017 0.022-0.091 | -- | 0.018 0.029-0.101 |  |  |
| 8.5 | -- | 0.033 0.082-0.212 |  | -- | 0.022 0.032-0.125 | -- | 0.032 0.083-0.209 |  |  |
| 9.0 | -- | 0.010 0.014-0.054 |  | -- | 0.017 0.021-0.091 | -- | 0.017 0.027-0.099 |  |  |
| 9.5 | -- | 0.033 0.081-0.212 |  | -- | 0.023 0.032-0.125 | -- | 0.032 0.081-0.209 |  |  |

Table S4 (refer to Table 6).

|  | Ψ^WE*^ | |  | Ψ^pE^ | | Ψ^cE^ | | Ψ^kE^ | |
| --- | --- | --- | --- | --- | --- | --- | --- | --- | --- |
|  | **(4c) **** | |  | **(3a)** | | **(3a)** | | **(3a)** | |
| **Age** | **Female** | **Male** |  | **Female** | **Male** | **Female** | **Male** | **Female** | **Male** |
| 0.5 | 0.011 0.006-0.058 | 0.010 0.008-0.054 |  | 0.067 0.032-0.322 | 0.037 0.023-0.186 | -- | -- | -- | 0.009 0.001-0.062 |
| 1.0 | 0.014 0.046-0.102 | 0.014 0.048-0.105 |  | 0.050 0.122-0.318 | 0.045 0.072-0.251 | -- | -- | 0.020 0.034-0.114 | 0.022 0.036-0.128 |
| 1.5 | 0.123 0.001-0.807 | 0.096 0.007-0.526 |  | 0.038 0.078-0.230 | 0.057 0.078-0.307 | -- | 0.035 0.018-0.178 | 0.020 0.033-0.114 | 0.025 0.038-0.141 |
| 2.0 | 0.007 0.062-0.088 | 0.010 0.097-0.137 |  | 0.028 0.082-0.195 | 0.047 0.102-0.286 | -- | 0.042 0.021-0.212 | 0.022 0.033-0.125 | 0.028 0.043-0.159 |
| 2.5 | 0.124 0.003-0.720 | 0.096 0.027-0.442 |  | 0.029 0.068-0.184 | 0.059 0.113-0.343 | -- | 0.072 0.038-0.340 | 0.024 0.032-0.129 | 0.034 0.043-0.159 |
| 3.0 | 0.007 0.085-0.111 | 0.010 0.144-0.184 |  | 0.032 0.083-0.211 | 0.056 0.131-0.351 | -- | 0.068 0.050-0.421 | 0.025 0.035-0.140 | 0.043 0.062-0.238 |
| 3.5 | 0.014 0.063-0.120 | 0.014 0.138-0.195 |  | 0.032 0.068-0.196 | 0.064 0.129-0.277 | -- | 0.092 0.044-0.386 | 0.024 0.029-0.131 | 0.044 0.064-0.242 |
| 4.0 | 0.109 0.009-0.553 | 0.084 0.067-0.403 |  | 0.036 0.081-0.224 | 0.059 0.132-0.361 | -- | 0.083 0.035-0.330 | 0.023 0.028-0.124 | 0.047 0.053-0.226 |
| 4.5 | 0.017 0.067-0.133 | 0.011 0.163-0.204 |  | 0.036 0.101-0.243 | 0.051 0.104-0.306 | -- | 0.069 0.029-0.292 | 0.022 0.028-0.124 | 0.042 0.053-0.226 |
| 5.0 | 0.090 0.007-0.505 | 0.051 0.030-0.252 |  | 0.024 0.055-0.152 | 0.046 0.085-0.268 | -- | 0.060 0.022-0.258 | 0.015 0.022-0.083 | 0.039 0.053-0.211 |
| 5.5 | 0.014 0.080-0.133 | 0.011 0.074-0.116 |  | 0.031 0.072-0.195 | 0.043 0.066-0.238 | -- | 0.052 0.016-0.215 | 0.017 0.026-0.095 | 0.037 0.044-0.200 |
| 6.0 | 0.013 0.029-0.081 | 0.020 0.052-0.131 |  | 0.023 0.039-0.134 | 0.036 0.047-0.196 |  |  | 0.013 0.017-0.074 | 0.033 0.039-0.178 |
| 6.5 | 0.111 0.004-0.640 | 0.085 0.010-0.445 |  | 0.032 0.056-0.187 | 0.034 0.036-0.177 |  |  | 0.017 0.020-0.091 | 0.032 0.032-0.167 |
| 7.0 | 0.014 0.017-0.076 | 0.019 0.045-0.123 |  | 0.024 0.030-0.129 | 0.029 0.025-0.152 |  |  | 0.014 0.014-0.073 | 0.032 0.028-0.163 |
| 7.5 | 0.093 0.004-0.578 | 0.052 0.019-0.259 |  | 0.034 0.047-0.187 | 0.027 0.018-0.138 |  |  | 0.017 0.017-0.091 | 0.030 0.023-0.154 |
| 8.0 | 0.012 0.014-0.064 | 0.013 0.047-0.100 |  | 0.024 0.026-0.128 | 0.023 0.012-0.121 |  |  | 0.014 0.012-0.074 | 0.031 0.020-0.157 |
| 8.5 | 0.014 0.038-0.093 | 0.020 0.039-0.122 |  | 0.035 0.042-0.187 | 0.021 0.010-0.110 |  |  | 0.018 0.015-0.093 | 0.029 0.017-0.149 |
| 9.0 | 0.044 0.001-0.406 | 0.064 0.008-0.360 |  | 0.024 0.023-0.127 | 0.018 0.006-0.098 |  |  | 0.014 0.010-0.075 | 0.031 0.015-0.157 |
| 9.5 | 0.015 0.034-0.094 | 0.020 0.035-0.116 |  | 0.036 0.040-0.188 | 0.016 0.005-0.089 |  |  | 0.018 0.014-0.094 | 0.029 0.012-0.148 |
| 10.0 | 0.037 0.002-0.306 | 0.027 0.016-0.191 |  | 0.024 0.022-0.127 | 0.013 0.003-0.080 |  |  | 0.015 0.010-0.076 | 0.030 0.011-0.159 |

*Abbreviations for origin and destination areas are: E=EDPS, W=WDPS, n=northern (EDPS), s=southern (EDPS), p=Prince William Sound (WDPS), c=Chiswell (WDPS), k=Kodiak (WDPS)

**Values indicate the best model based on AICc (see Table 2); if more than one model is listed, model averaging was used with models in the order listed, with model weights in brackets.
